# Evaluation of foods, drinks and diets in the Netherlands according to the degree of processing for nutritional quality, environmental impact and food costs

**DOI:** 10.1186/s12889-022-13282-x

**Published:** 2022-05-03

**Authors:** Reina E. Vellinga, Marieke van Bakel, Sander Biesbroek, Ido B. Toxopeus, Elias de Valk, Anne Hollander, Pieter van ’t Veer, Elisabeth H. M. Temme

**Affiliations:** 1 Centre for Nutrition, Prevention and Health Services, National Institute for Public Health and the Environment (RIVM), Antonie van Leeuwenhoeklaan 9, Bilthoven, 3721 MA The Netherlands; 2 Division of Human Nutrition and Health, Wageningen University, P.O. Box 17, 6700 AA Wageningen, The Netherlands

## Abstract

**Objective:**

This study investigates nutritional quality, environmental impact and costs of foods and drinks and their consumption in daily diets according to the degree of processing across the Dutch population.

**Design:**

The NOVA classification was used to classify the degree of processing (ultra-processed foods (UPF) and ultra-processed drinks (UPD)). Food consumption data were derived from the Dutch National Food Consumption Survey 2012–2016. Indicators assessed were nutritional quality (saturated fatty acids (SFA), sodium, mono and disaccharides (sugar), fibre and protein), environmental impact (greenhouse gas (GHG) emissions and blue water use) and food costs.

**Setting:**

The Netherlands.

**Participants:**

Four thousand three hundred thirteen Dutch participants aged 1 to 79 years.

**Results:**

Per 100 g, UPF were more energy-dense and less healthy than unprocessed or minimally processed foods (MPF); UPF were associated with higher GHG emissions and lower blue water use, and were cheaper. The energy and sugar content of UPD were similar to those of unprocessed or minimally processed drinks (MPD); associated with similar GHG emissions but blue water use was less, and they were also more expensive. In the average Dutch diet, per 2000 kcal, ultra-processed foods and drinks (UPFD) covered 29% (456 g UPF and 437 g UPD) of daily consumption and 61% of energy intake. UPFD consumption was higher among children than adults, especially for UPD. UPFD consumption determined 45% of GHG emissions, 23% of blue water use and 39% of expenses for daily food consumption. UPFD consumption contributed 54% to 72% to daily sodium, sugar and SFA intake.

**Conclusions:**

Compared with unprocessed or minimally processed foods and drinks, UPF and UPD were found to be less healthy considering their high energy, SFA, sugar and sodium content. However, UPF were associated higher GHG emissions and with less blue water use and food costs. Therefore daily blue water use and food costs might increase if UPF are replaced by those unprocessed or minimally processed. As nutritional quality, environmental impacts and food costs relate differently to the NOVA classification, the classification is not directly applicable to identify win–win-wins of nutritional quality, environmental impact and costs of diets.

**Supplementary Information:**

The online version contains supplementary material available at 10.1186/s12889-022-13282-x.

## Background

Providing healthy and sustainable diets is one of the major challenges of this century. Considering global warming and the rise of nutrition-related non-communicable diseases (NCDs) [1], it is essential to identify, understand, and influence key drivers that contribute to unhealthy and unsustainable diets. In the last few decades, the global nutritional transition is characterized by a shift towards the consumption of ultra-processed foods (UPF) at the expense of basic, unprocessed foods [2, 3]. UPF are mostly or entirely created from substances extracted from foods or derived from food constituents and are transformed into unrecognizable, ready-to-eat foods that contain additives and high amounts of energy, sugar, fat and salt [4]. In contrast, unprocessed or minimally processed foods and drinks are those that are either fresh or slightly altered to increase food safety, accessibility or palatability.

Food processing should be an integral part of a sustainable food system [5, 6]. For instance, food processing makes food safer, enables preservation of foods, helps to overcome seasonal gaps, enables nose-to-tail consumption and encourages reuse of materials [6]. On the other hand, food processing steps such as manufacturing, packaging and distribution, contribute to GHG emissions [7]. Moreover, considerable amounts of energy, water and packaging materials are used for food processing. The latter significantly contributes to the plastic waste stream entering marine ecosystems [7].

Processes and ingredients that are used to manufacture UPF make them highly convenient for consumers and highly profitable for manufacturers [4]. Over the past years, it has been argued that unhealthy foods are less expensive compared with healthy foods while the price gap between them is growing [8]. Considering that food prices are an important determinant of food choices and nutritious diets, affordability of ultra-processed foods seems inevitably linked to its consumption, which may have implications for public health, health inequalities and food security, among others [9].

Recent studies link UPF with adverse health outcomes. Higher availability or consumption of UPF is associated with increased risk of overweight, obesity, cardiovascular diseases (CVD), cancer and all-cause mortality [10–12]. In food-based dietary guidelines, several countries recommend reducing UPF consumption (for example, in Brazil [13] and Canada [14]) or have set targets to reduce UPF consumption (for example, by 20% in France by 2022 [15]). Existing literature on UPF has primarily focused on nutrient profiles or health outcomes. Less is known about the association between UPF and environmental impact or food costs.

The NOVA classification is often used to categorize foods according to the degree of processing [4]. It could potentially be used to distinguish nutritional quality, environmental impact and cost of diets. If those indicators were consistently different in ultra-processed foods and drinks (UPFD) compared with unprocessed or minimally processed foods and drinks (MPFD), this would facilitate a win–win-win scenario for the transition towards a healthy and sustainable diet. Therefore, this study examines the nutritional quality (via energy, saturated fatty acids (SFA), sodium, fibre, mono and disaccharides (sugar) and protein), environmental impact (via GHG emissions and blue water use) and food costs for UPFD compared with MPFD, as well as their consumption across a representative Dutch population.

## Methods

### Population and dietary data

Data for 4,313 Dutch children and adults aged 1 to 79 years were derived from the Dutch National Food Consumption Survey (DNFCS) 2012–2016 [16]. Food consumption data was obtained using two 24-h non-consecutive dietary recalls and reported in Globodiet software (IARC©; former EPIC-Soft) [17]. Background information such as date of birth, urbanisation level and educational level was collected by the market research agency who was responsible for the representativeness. Information on body composition was gathered in different ways depending on age: body weight and height of 1–15-year-olds were measured, for 16–70-year-olds they were self-reported and body weight of < 70-year-olds was measured by a trained dietician. Height was not measured for adults aged 71–79-years due to practical reasons. Body Mass Index (BMI) was calculated as the average body weight (in kg) divided by average height (in m) squared (kg/m^2^). A full explanation and description of this survey are reported elsewhere [16]. For the current study, participants were classified into subgroups based on age (1–3, 4–8, 9–18, 19–30, 31–50 and 51–79 year-olds), weight status (underweight (BMI < 18.5 kg/m^2^), normal weight (BMI 18.5– < 25 kg/m^2^), overweight (BMI 25– < 30 kg/m^2^), and obese (BMI ≥ 30 kg/m^2^), level of education, and degree of urbanization. The level of education was classified as low (primary education, lower vocational education, advanced elementary education), moderate (intermediate vocational education, higher secondary education) or high (higher vocational education and university). The educational level concerned the participants’ highest completed educational level or, in the case of participants under the age of 19 years, of the head of household. The degree of urbanization was classified as hardly urbanized (fewer than 1,000 addresses/km^2^), moderately urbanized (1000–1500 addresses/km^2^) and highly urbanized (1,500 or more addresses/km^2^) [16].

### Degree of food processing

The NOVA food classification system was applied to determine the degree of food processing [4]. NOVA categorizes foods and drinks according to the nature, extent, and purpose of the industrial processing they undergo. The classification distinguishes four categories: unprocessed or minimally processed foods, processed culinary ingredients, processed foods, and ultra-processed foods, which are described in detail elsewhere [4]. In the current study, foods and drinks were classified into separate categories. Via facet descriptions from Globodiet, all unique foods and drinks reported by participants were identified and systematically categorized into one of the four NOVA categories. Ingredients of composite dishes were individually reported. The following facets descriptions were used: conservation method (e.g. fresh, pasteurization, canned, frozen); production (e.g. industrial, ready-to-eat, fresh); medium (e.g. in oil, in brine, in syrup); salt content (e.g. salted or not salted); sugar content (e.g. not sweetened or sweetened with sugar and/or artificial sweeteners) and where appropriate consistency/shape (e.g. powder, liquid, sliced). Food groups were based on Globodiet. Food group-specific categorization can be found in Supplemental Table S1. In short, fresh or plain foods and drinks or slightly altered (dried, frozen, steamed) were classified as unprocessed or minimally processed foods (MPF) or drinks (MPD) such as plain yoghurt, rice, coffee and tea. Vegetable oils, butter and other animal fats, and sugar were categorized as processed culinary ingredients. Fresh or slightly altered foods combined with processed culinary ingredients were classified as processed foods or drinks (e.g. tuna in oil, salted nuts). Foods and drinks that were either ready-to-eat, industrially prepared, contained many additives, emulsifiers and/or other comparable formulations/ingredients were classified as ultra-processed (e.g. fruity dairy drinks, confectionery, margarine). All bread was classified as ultra-processed since most bread is industrially prepared and contains food additives. Alcoholic drinks are not classified according to the NOVA classification. In the current study, wine, cider and beer were classified as processed as they are produced by fermentation of unprocessed foods. Other spirits and liquors (e.g. gin or whisky) were classified as ultra-processed. A research dietician cross-checked the classification and provided expert judgement.

### Nutritional quality

Foods and drinks from the DNFCS 2012–2016 were linked to food composition data of the Dutch Food Composition Database (NEVO online version 2016/5.0) in order to estimate daily intake of energy, SFA, sodium, mono and disaccharides (sugars), fibre and protein [18]. In addition to often assessed nutrients (e.g. energy, SFA, sodium, sugar and fibre) that associate with UPFD consumption, protein is of importance since proteins plays an important role in the transition towards a sustainable diet. Mono and disaccharides were assessed since free or added sugar are not included in the Dutch food composition table (NEVO-online version 2016/5.0).

### Environmental impact

The environmental impacts of foods were evaluated for Greenhouse gas (GHG) emissions (in kg CO_2_-eq) and blue water use (in m^3^). Blue water use is also referred to as irrigation water. Data on environmental impact were derived from the Dutch Life Cycle Assessment (LCA) food database [19]. In a previous study in which we applied the LCA Food database we showed that the correlation between GHG emissions and other environmental indicators is generally high, except for blue water use [20]. Therefore, this study examines, besides GHG emission, blue water use since this indicator focusses on other important foods which are ignored when solely focussing on GHG emissions. In short, environmental impacts were based on LCA methodology, which quantified the environmental impact through the foods’ entire life cycle. LCAs had an attributional approach and hierarchical perspective and were performed following the ISO 14040 and 14,044 guidelines. A time horizon of 100 years was used, and GHG emissions were recalculated following Intergovernmental Panel on Climate Change (IPCC) guidelines (2006) [21]. Economic allocation was applied when production processes led to more than one food product, except for milk, for which bio-physical allocation was used. The functional unit used was 1 kg of prepared food or drink on the plate, and converted to per 100 g. The LCA food database provided primary data for 265 foods and drinks, which cover 75% of total amount of food intake. These foods were previously selected based on frequency of consumption in the DNFCS and variation in types of food. The environmental impact of foods and beverages for which primary data were not available but that were consumed in the DNFCS 2012–2016 were matched with similar foods. The same methodology was applied in a previous study [20]. In short, foods were matched by expert judgement of a panel of scientists and were based on similarities in types of food, production systems and ingredient composition. For composite dishes, standardized recipes from the Dutch Food composition table (NEVO-online version 2016/5.0) were used where available and if not available, recipes were based on label information. More detailed information on the use of the database can be found elsewhere [19, 20].

### Food costs

The Dutch food cost database was used to estimate food costs. A detailed description of the database can be found elsewhere [22]. Briefly, retail food prices (*n* = 902) of the lowest, non-promotional price were collected from a high segment supermarket (Albert Heijn) and a discount supermarket (Lidl) during July and August 2017 in Amsterdam, the Netherlands. Prices were adjusted for the weight of packaging, preparation (shrinkage/gain) and waste and expressed in € per 100 g edible portion. Eight hundred thirty-nine food prices were directly linked to food composition data of the Dutch Food Composition Database (NEVO-online version 2016/5.0) and covered 62% of the total amount of food intake [18]. Remaining foods were matched to similar foods based on similarities in product, brand, (relative) price and ingredient composition. For composite dishes, standardized recipes from the Dutch food composition table (NEVO-online version 2016/5.0) were used.

### Data analysis

Descriptive statistics were applied to characterize the nutritional and environmental indicators and costs for foods and drinks (per 100 g) reported by DNFCS 2012–2016, according to the degree of processing. Primary data was used to characterize environmental impact and costs according to the degree of processing. Notable differences in characteristics between foods and drinks per 100 g according to their degree of processing were reported based on mean and 95%CI. Daily average consumption of UPFD, UPF and UPD was calculated over two consumption days and expressed in weight (g) per 2000 kcal. The outcomes were standardized in order to assess the relative contribution of food intake according to degree of processing towards the total dietary intake. Mann–Whitney U test or Kruskal–Wallis test for non-normally distributed data and ANOVA for normal distributed data were applied to examine differences in UPFD consumption across population subgroups. Nutritional quality (energy, SFA, sodium, sugar, fibre and protein), environmental impact (GHG emissions and blue water use) and food costs for total diet and according to degree of processing were calculated over two consumption days and standardized to 2000 kcal per day and were reported for total diet and according to degree of processing. Wilcoxon signed rank test for non-normally distributed data and paired t-test for normal distributed data were used to assess whether the nutritional quality, environmental impacts and food costs of the consumption of culinary processed ingredients, processed foods and drinks, and UPF and UPD differs from those of unprocessed or minimally processed foods and drinks. Descriptive statistics were reported as mean, 95% confidence interval (95%CI), 25^th^ percentile, 50^th^ percentile and 75^th^ percentile (P25, P50, P75). Reported values were weighted for demographic properties, season, and combination of both consumption days (week or weekend). A sensitivity analysis was performed with alternations made in the food classification for bread (processed instead of ultra-processed). The statistical analysis was performed using SAS software, version 9.4 (SAS Institute Inc., Cary, NC, USA). A two-sided *p*-value of < 0.05 was considered statistically significant.

## Results

### Foods and drinks classified according to NOVA

**Fig. 1:**
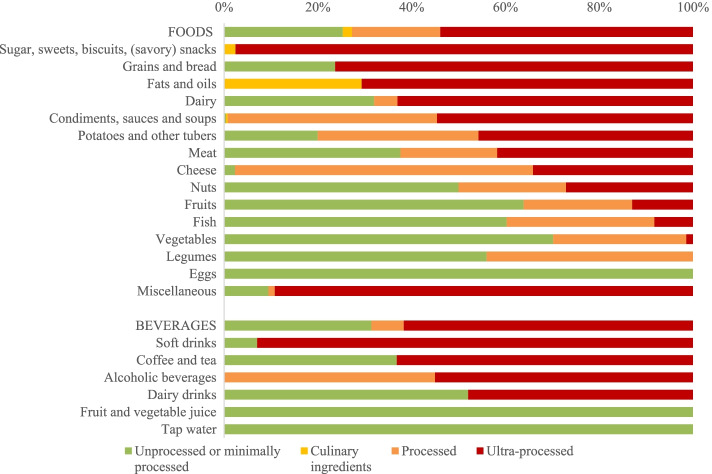
Percentage of foods and drinks according to NOVA-categories for foods and drinks consumed in DNFCS 2012–2016 by food groups

Around half to two-thirds of the foods (54%) and drinks (62%) identified in DNFCS 2012–2016 were categorized as ultra-processed foods (UPF) or drinks (UPD) (Fig. 1). Approximately a quarter of foods (25%) and one-third of drinks (31%) were classified as unprocessed or minimally processed foods (MPF) or drinks (MPD). In the food groups ‘Sugar, sweets and (savoury) snacks’ (98%), ‘Soft drinks’ (93%) ‘Grains and breads’ (76%), and ‘Fats and oils’ (71%), the majority of foods were classified as UPF or UPD. The food groups ‘Eggs’ (0%), ‘Legumes’ (0%), ‘Vegetables’ (1%), ‘Fish’ (8%), ‘Fruits’ (13%), ‘Tap water’ (0%) and ‘Fruit and vegetable juice’ (0%) contained a low or no share of UPF or UPD.

### Characteristics of ultra-processed foods and drinks

UPF contained around double the amount of energy (313 vs 150 kcal/100 g (+ 109%)), triple the mono and disaccharides (16.1 vs 4.9 g/100 g (+ 229%)) and SFA (5.4 vs 1.9 g/100 g (+ 184%)), and four times the sodium (478 vs 126 mg/100 g (+ 279%)) compared with MPF (Table 1). UPF contained reasonably similar amounts of protein (7.1 vs 8.9 g/100 g) and fibre (2.3 vs 2.7 g/100 g) compared with MPF. UPD had a similar energy (67 vs 75 kcal/100 g) and mono- and disaccharides (8.7 vs 7.3 g/100 g) content compared with MPD.

**Table 1:** The average nutritional quality, environmental impact and costs aspects per 100 g foods and drinks in the DNFCS 2012–2016 by degree of processing

	**Energy (kcal)/100 g**						**Mono and disaccharides (g)/100 g**						
	*N*	Mean	(95%CI)	P25, P50, P75			*N*	Mean	(95%CI)		P25, P50, P75		
**All**	2159	243	(235, 251)	71	211	368	2159	10.7	(10.0, 11.5)		0.4	3.1	11.9
**Foods**													
Unprocessed or minimally processed (MPF)	481	150	(136, 164)	36	105	189	481	4.9	(4.0, 5.9)		0.0	1.6	4.5
Processed culinary ingredients	37	664	(575, 752)	396	737	899	37	23.8	(10.2, 37.4)		0.0	0.2	28.2
Processed	358	249	(228, 271)	83	194	355	358	3.7	(2.8, 4.5)		0.0	0.6	4.0
Ultra-processed (UPF)	1022	313	(303, 323)	194	302	413	1022	16.1	(14.9, 17.4)		1.3	5.3	25.3
**Drinks**													
Unprocessed or minimally processed (MPD)	82	75	(50, 99)	35	47	68	82	7.3	(5.3 9.4)		2.1	5.1	9.7
Processed	18	83	(59, 107)	47	66	111	18	4.9	(2.4, 7.5)		0.9	3.1	6.0
Ultra-processed (UPD)	161	67	(54, 80)	24	40	66	161	8.7	(7.2, 10.3)		3.9	7.4	10.8
	**Sodium (mg)/100 g**						**Saturated fatty acids (g)/100 g**						
	*N*	Mean	(95%CI)	P25, P50, P75			*N*	Mean	(95%CI)		P25, P50, P75		
**All**	2159	325	(284, 365)	10	74	400	2159	4.4	(4.1, 4.8)		0.1	1.2	6.2
**Foods**													
Unprocessed or minimally processed (MPF)	481	126	(91, 160)	5	25	75	481	1.9	(1.5, 2.3)		0.0	0.2	2.0
Processed culinary ingredients	37	62	(19, 105)	0	0	10	37	19.2	(12.5, 25.9)		1.3	14.3	27.2
Processed	358	398	(334, 463)	26	232	600	358	6.5	(5.5, 7.5)		0.1	2.6	9.7
Ultra-processed (UPF)	1022	478	(398, 558)	50	240	524	1022	5.4	(4.9, 5.8)		0.6	2.6	8.3
**Drinks**													
Unprocessed or minimally processed(MPD)	82	51	(29, 72)	2	22	39	82	0.9	(0.4, 1.5)		0.0	0.0	1.0
Processed	18	6	(2, 10)	2	3	4	18	0.0	(0.0, 0.0)		0.0	0.0	0.0
Ultra-processed (UPD)	161	19	(14, 23)	1	5	40	161	0.3	(0.1, 0.6)		0.0	0.0	0.0
	**Fibre (g)/100 g**						**Protein (g)/100 g**						
	*N*	Mean	(95%CI)	P25, P50, P75			*N*	Mean	(95%CI)		P25, P50, P75		
**All**	2159	2	(1.8, 2.1)	0	0.7	2.5	2159	7.1	(6.7, 7.4)		0.8	3.8	10.8
**Foods**													
Unprocessed or minimally processed (MPF)	481	2.7	(2.3, 3.1)	0.0	1.6	3.0	481	8.9	(8.1, 9.7)		1.1	3.9	18.3
Processed culinary ingredients	37	0.2	(0.1, 0.5)	0.0	0.0	0.0	37	0.3	(0.1, 0.6)		0.0	0.0	0.3
Processed	358	1.2	(1.0, 1.5)	0.0	0.1	1.7	358	9.5	(8.3, 10.6)		0.7	2.8	19.5
Ultra-processed (UPF)	1022	2.3	(2.1, 2.5)	0.2	1.3	3.4	1022	7.1	(6.7, 7.5)		2.1	5.8	10.0
**Drinks**													
Unprocessed or minimally processed (MPD)	82	0.4	(0.2, 0.5)	0.0	0.2	0.4	82	2.5	(1.3, 3.6)		0.4	1.0	2.2
Processed	18	0.1	(0.0, 0.2)	0.0	0.0	0.3	18	0.2	(0.1, 0.3)		0.0	0.2	0.4
Ultra-processed (UPD)	161	0.4	(0.1, 0.6)	0.0	0.0	0.2	161	1.0	(0.6, 1.4)		0.0	0.1	1.3
	**GHG emissions (kg CO**_**2**_**-eq)/100 g**						**Blue water use (m**^**3**^**)/100 g**						
	*N*	Mean	(95%CI)	P25, P50, P75			*N*	Mean	(95%CI)		P25, P50, P75		
**All**	265	0.54	(0.44, 0.64)	0.14	0.25	0.62	265	0.021	(0.013, 0.029)		0.002	0.006	0.012
**Foods**													
Unprocessed or minimally processed (MPF)	106	0.55	(0.35, 0.74)	0.12	0.21	0.52	106	0.033	(0.017, 0.049)		0.004	0.007	0.017
Processed culinary ingredients	7	0.57	(0.10, 1.04)	0.11	0.50	1.22	7	0.060	(0.070, 0.191)		0.001	0.010	0.018
Processed	20	0.84	(0.52, 1.16)	0.27	0.85	1.10	20	0.036	(0.010, 0.082)		0.005	0.010	0.012
Ultra-processed (UPF)	98	0.62	(0.49, 0.75)	0.22	0.42	0.64	98	0.008	(0.007, 0.010)		0.004	0.006	0.011
**Drinks**													
Unprocessed or minimally processed (MPD)	13	0.11	(0.06, 0.15)	0.04	0.14	0.15	13	0.008	(0.002, 0.019)		0.001	0.002	0.004
Processed	4	0.18	(0.06, 0.30)	0.14	0.21	0.22	4	0.007	(0.001, 0.013)		0.005	0.009	0.009
Ultra-processed (UPD)	17	0.10	(0.05, 0.14)	0.05	0.06	0.07	17	0.002	(0.001, 0.002)		0.001	0.001	0.002
	**Food costs ( €)/100 g**												
	*N*	Mean	(95%CI)	P25, P50, P75									
**All**	993	0.65	(0.57, 0.74)	0.19	0.37	0.80							
**Foods**													
Unprocessed or minimally processed (MPF)	256	0.97	(0.66, 1.29)	0.20	0.48	1.09							
Processed culinary ingredients	12	0.26	(0.14, 0.38)	0.12	0.20	0.38							
Processed	163	0.70	(0.60, 0.79)	0.23	0.54	0.98							
Ultra-processed (UPF)	458	0.55	(0.51, 0.59)	0.24	0.40	0.72							
**Drinks**													
Unprocessed or minimally processed (MPD)	31	0.15	(0.07, 0.24)	0.08	0.11	0.13							
Processed	9	0.33	(0.25, 0.41)	0.25	0.36	0.40							
Ultra-processed (UPD)	64	0.37	(0.23, 0.52)	0.09	0.13	0.36							

UPF were associated with slightly higher GHG emissions (0.62 vs 0.55 kg CO_2_-eq/100 g (+ 12%)) but less usage of blue water (0.008 vs 0.033 m^3^/100 g (-97%)) compared with MPF. Underlaying food groups showed a large variation in average environmental impact, e.g. GHG emissions were on average 0.19 kg CO_2_-eq/100 g for unprocessed or minimally processed vegetables while 2.75 kg CO_2_-eq/100 g for unprocessed or minimally processed meat. UPD were associated with similar GHG emissions (0.11 vs 0.10 kg CO_2_-eq/100 g) but less blue water use (0.002 vs 0.008 m^3^/100 g (-75%)) than MPD. UPF were almost half as expensive as MPF (€0.55 vs €0.97/100 g (-43%)). UPD cost two times more (€0.37 vs €0.15/100 g (+ 147%)) compared with MPD.

### Ultra-processed foods and drinks in daily diets

**Fig. 2:**
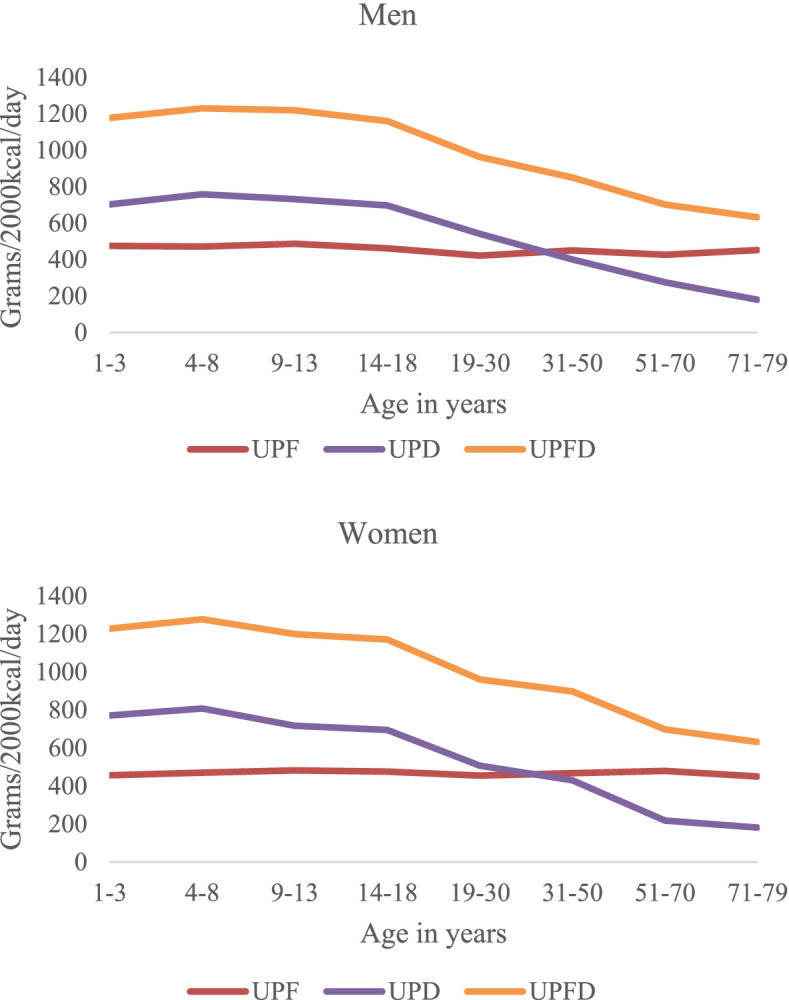
The daily average ultra-processed foods and drink consumption in grams per 2000 kilocalories for Dutch men and women aged 1 to 79 years according to different age groups

The Dutch population consumed a daily absolute average of 3053 g (2126 kcal) of foods and drinks, of which 925 g UPFD (478 g UPF and 477 g UPD). The absolute daily average UPFD consumption was 743 g for 1–3-year-olds, 1014 g for 4–8-year-olds, 1230 g for 9–13-year-olds, 1259 g for 14–18-year-olds, 1091 g for 19–30-year-olds, 959 g for 31–50-year-olds, 737 g for 51–70-year-olds and 617 g for 71–79-year-olds. Figure 2shows the daily consumption of UPF and UPD by age, in grams per 2000 kcal. Per 2000 kcal, the daily average UPFD consumption was 893 g (456 g UPF and 437 g UPD) and did not differ between men (889 g/2000 kcal) and women (898 g/2000 kcal) (*p* > 0.05) (Table 2). Daily UPFD consumption differs significantly between age groups (*p* < 0.001). Children and teenagers up to 18 years consumed, almost twice as much UPFD (approximately 1200 g/2000 kcal) compared with adults and older adults aged 51 to 79 years (ranging between 632 g/2000 kcal to 700 g/2000 kcal). Adults aged 19 to 30 years and 31 to 50 years consumed 962 g and 874 g UPFD per 2000 kcal, respectively. Consumption of UPF ranged from 438 to 485 g/2000 kcal for all age groups. Children and teenagers consumed more UPD (approximately 700 g/2000 kcal) than adults aged 19 to 50 years old (415 to 525 g/2000 kcal) and adults aged 51 to 79 years old (ranging between 180 to 247 g/2000 kcal).

**Table 2:** The average and distribution of the consumption of ultra-processed foods and drinks in grams per 2000 kilocalories for total Dutch population and subgroups of the population

	*N*	*Total UPFD (g)/ 2000 kcal *^**a**^						*Ultra-processed foods (UPF) (g)/ 2000 kcal*						*Ultra-processed drinks (UPD) (g)/ 2000 kcal*					
	%	Mean	(95%CI)	P25, P50, P75			*P*-value	Mean	(95%CI)	P25, P50, P75			*P*-value	Mean	(95%CI)	P25, P50, P75			*P*-value
**All**	100	893	(879, 907)	547	790	1156		456	(452, 461)	353	439	533		437	(423, 450)	93	314	669	
**Gender**																			
Male	50	889	(870, 907)	551	788	1143		444	(439, 450)	353	434	516		444	(426, 462)	103	332	664	
Female	50	898	(877, 918)	544	794	1172		469	(461, 476)	354	446	554		429	(410, 449)	75	286	672	
**Age(y)**							***						***						***
1–3	16	1202	(1159, 1246)	799	1145	1494		466	(453, 478)	364	440	536		737	(695, 778)	361	650	1007	
4–8	12	1252	(1217, 1288)	953	1233	1477		471	(460, 482)	388	459	535		781	(746, 817)	483	760	1025	
9–13	12	1209	(1175, 1243)	936	1193	1460		485	(474, 495)	404	468	547		725	(691, 758)	465	699	981	
14–18	12	1165	(1124, 1206)	817	1097	1455		469	(459, 479)	391	461	538		696	(656, 737)	350	615	990	
19–30	12	962	(921, 1003)	622	853	1209		438	(425, 450)	350	419	503		524	(483, 565)	173	406	755	
31–50	12	874	(834, 914)	544	768	1106		459	(446, 472)	350	445	541		415	(377, 453)	97	292	617	
51–70	12	700	(669, 730)	449	628	857		453	(437, 468)	335	429	541		247	(220, 274)	0	137	342	
71–79	12	632	(607, 656)	436	570	757		451	(438, 465)	345	438	533		180	(161, 200)	0	113	276	
**BMI**^**b**^							***						**						***
Underweight	5	1104	(1044, 1165)	794	1091	1375		477	(458, 495)	373	470	576		627	(566, 688)	273	568	940	
Normal weight	56	924	(905, 942)	571	833	1206		450	(444, 456)	351	432	524		473	(456, 491)	128	371	712	
Overweight	18	878	(846, 910)	558	768	1113		462	(451, 473)	358	442	543		416	(385, 446)	89	302	658	
Obese	8	907	(852, 962)	542	770	1161		462	(444, 480)	349	441	545		445	(393, 497)	74	274	649	
**Education level**							***						**						***
Low	19	871	(838, 903)	541	733	1144		457	(446, 468)	350	440	534		414	(382, 445)	60	273	638	
Moderate	37	939	(916, 962)	585	839	1206		458	(451, 466)	355	436	533		481	(458, 503)	128	360	751	
High	44	850	(830, 871)	518	754	1096		454	(447, 461)	356	443	533		397	(378, 416)	82	270	610	
**Degree of urbanization**							**						**						
Low	34	876	(856, 896)	544	777	1117		443	(436, 450)	345	428	522		432	(413, 451)	102	312	664	
Moderate	20	898	(868, 928)	552	801	1162		465	(455, 476)	368	445	532		432	(404, 461)	85	315	655	
High	46	916	(891, 942)	547	814	1202		470	(462, 479)	363	448	547		446	(422, 470)	88	314	693	

^a^ Uncorrected energy intake was 1236 kcal for 1–3 year old, 1644 kcal for 4–8 years old, 2052 kcal for 9 to 13 years old, 2207 kcal for 14 to 18 years old, 2317 kcal for 19 to 30 years old, 2250 kcal for 31 to 50 years old, 2139 kcal for 51 to 70 years old and 1966 kcal for 71 to 79 years old

^b^ 12% missings;

^*^ < 0.05, ** < 0.01 *** < 0.001

There were significant differences overall by subgroups of education level and degree of urbanization (Table 2), ranging around 4–9% between the subgroups. Participants with a moderate education level (939 (95%CI 916, 962) g/2000 kcal) consumed 89 g more UPFD compared with higher educated participants (850 (95%CI 830, 871) g/2000 kcal) and 68 g more compared with lower educated participants (871 (95%CI 838,903) g/2000 kcal) (*p* < 0.001). Participants living in low urbanized areas consumed 916 (95%CI 891, 942) g/2000 kcal UPFD, and consumed 40 or 20 g UPFD more than those living in highly or moderately urbanized areas, 876 (95%CI 856, 896) g/ 2000 kcal and 898 (95%CI 868, 928) g/ 2000 kcal respectively (*p* < 0.01).

### Nutritional quality, environmental impact and food costs

Although there was a statistically significant difference observed between UPF and MPF consumption, their consumption was more or less similar with 442 g/2000 kcal and 456 g/2000 kcal, respectively for UPF and MPF. Energy intake from UPF was almost three times higher at 1107 kcal (55%)compared with 372 kcal (19%) from MPF (*p* < 0.001). Per 2000 kcal, UPF consumption contributed most towards daily intake of sodium (1596 mg, 70%), fibre (11.1 g, 58%), SFA (15.3 g, 54%), protein (33 g, 44%) and mono and disaccharides (42 g, 40%) (Table 3). MPF consumption contributed less to daily nutrient intake, ranging between 7% (for sodium) and 37% (for fibre). The consumption of UPD (437 g/2000 kcal) was around three times lower than the consumption of MPD (1510 g/2000 kcal) (*p* < 0.001), contributed 6% to daily energy intake and determined 25 g (24%) of daily sugar intake.

**Table 3:** The average consumption, nutritional quality, environmental impact and food costs aspects per 2000 kilocalories consumed foods and drinks by degree of processing for the Dutch population

	**Weight (g)**						**Energy (kcal)**					
	Mean	(95%CI)	P25, P50, P75			*P*-value^1^	Mean	(95%CI)	P25, P50, P75			*P*-value
**All**	3039	(3006, 3073)	2258	2801	3499		2000	(2000, 2000)	2000	2000	2000	
**Foods**												
Unprocessed or minimally processed (MPF)	442	(434, 450)	243	396	576		372	(366, 379)	215	346	489	
Processed culinary ingredients	11	(10, 11)	1	6	16	***	63	(61, 65)	7	40	93	***
Processed	78	(76, 80)	34	65	108	***	177	(173, 181)	83	155	246	***
Ultra-processed (UPF)	456	(452, 461)	353	439	533	***	1107	(1099, 1115)	928	1117	1292	***
**Drinks**												
Unprocessed or minimally processed (MPF)	1510	(1477, 1542)	770	1304	1993		96	(93, 99)	11	71	144	
Processed	106	(100, 113)	0	0	130	***	62	(59, 66)	0	0	84	***
Ultra-processed (UPD)	437	(423, 450)	93	314	669	***	122	(118, 127)	7	76	186	***
	**Mono and disaccharides (g)**						**Sodium (mg)**					
	Mean	(95%CI)	P25, P50, P75			*P*-value	Mean	(95%CI)	P25, P50, P75			*P*-value
**All**	105	(104, 107)	77	102	130		2295	(2275, 2315)	1850	2189	2648	
**Foods**												
Unprocessed or minimally processed (MPF)	19	(19, 20)	6	15	28		169	(164, 175)	57	114	207	
Processed culinary ingredients	5	(4, 5)	0	0	5	***	3	(2, 3)	0	0	0	***
Processed	2	(1, 2)	0	0	2	***	389	(379, 398)	156	320	538	***
Ultra-processed (UPF)	42	(41, 42)	26	39	54	***	1596	(1578, 1614)	1198	1521	1922	***
**Drinks**												
Unprocessed or minimally processed (MPF)	12	(12, 12)	1	9	18		89	(86, 92)	24	62	125	
Processed	1	(1, 1)	0	0	0	***	4	(4, 5)	0	0	6	***
Ultra-processed (UPD)	25	(24, 26)	0	14	37	***	45	(43, 47)	1	17	61	***
	**SFA (g)**						**Fibre (g)**					
	Mean	(95%CI)	P25, P50, P75			*P*-value	Mean	(95%CI)	P25, P50, P75			*P*-value
**All**	28.2	(28.0, 28.4)	23.3	28.0	32.4		19.0	(18.9, 19.2)	15.0	18.4	22.4	
**Foods**												
Unprocessed or minimally processed (MPF)	3.1	(3.0, 3.2)	0.9	2.1	4.2		6.4	(6.2, 6.5)	3.0	5.5	8.5	
Processed culinary ingredients	1.6	(1.5, 1.7)	0.0	0.4	1.6	***	0.0	(0.0, 0.0)	0.0	0.0	0.0	***
Processed	6.4	(6.2, 6.6)	2.4	5.3	9.2	***	0.8	(0.8, 0.9)	0.0	0.1	1.0	***
Ultra-processed (UPF)	15.3	(15.1, 15.5)	11.3	15.1	18.8	***	11.1	(11.0, 11.2)	8.5	10.9	13.4	***
**Drinks**												
Unprocessed or minimally processed (MPF)	1.4	(1.4, 1.5)	0.0	0.5	2.2		0.2	(0.2, 0.2)	0.0	0.0	0.3	
Processed	0.0	(0.0, 0.0)	0.0	0.0	0.0	***	0.2	(0.2, 0.2)	0.0	0.0	0.0	***
Ultra-processed (UPD)	0.4	(0.4, 0.4)	0.0	0.0	0.1	***	0.3	(0.3, 0.3)	0.0	0.0	0.2	***
	**Protein (g)**						**GHG emissions (kg CO**_**2**_**-eq)**					
	Mean	(95%CI)	P25, P50, P75			*P*-value	Mean	(95%CI)	P25, P50, P75			*P*-value
**All**	75.3	(74.8, 75.9)	63.1	72.6	85.2		4.72	(4.69, 4.77)	3.80	4.50	5.41	
**Foods**												
Unprocessed or minimally processed (MPF)	23.2	(22.7, 23.7)	10.4	20.0	32.1		1.40	(1.36, 1.43)	0.59	1.09	1.85	
Processed culinary ingredients	0.0	(0.0, 0.0)	0.0	0.0	0.0	***	0.05	(0.05, 0.05)	0.00	0.02	0.06	***
Processed	11.1	(10.8, 11.4)	4.5	9.3	15.6	***	0.59	(0.57, 0.60)	0.26	0.50	0.82	***
Ultra-processed (UPF)	33.1	(32.8, 33.4)	25.4	32.1	39.5	***	1.70	(1.68, 1.73)	1.08	1.53	2.14	***
**Drinks**												
Unprocessed or minimally processed (MPF)	6.0	(5.8, 6.1)	1.1	3.8	9.2		0.55	(0.53, 0.56)	0.24	0.46	0.77	
Processed	0.3	(0.3, 0.3)	0.0	0.0	0.1	***	0.13	(0.12, 0.13)	0.00	0.00	0.17	***
vUltra-processed (UPD)	1.7	(1.6, 1.8)	0.0	0.1	2.0	***	0.32	(0.31, 0.33)	0.05	0.21	0.46	***
	**Blue water use (m**^**3**^**)**						**Food costs (€)**					
	Mean	(95%CI)	P25, P50, P75			*P*-value	Mean	(95%CI)	P25, P50, P75			*P*-value
**All**	0.139	(0.137, 0.141)	0.084	0.120	0.171		4.32	(4.27, 4.36)	3.31	4.09	4.98	
**Foods**												
Unprocessed or minimally processed (MPF)	0.048	(0.046, 0.049)	0.018	0.035	0.061		1.32	(1.29, 1.35)	0.60	1.09	1.75	
Processed culinary ingredients	0.007	(0.006, 0.007)	0.000	0.001	0.005	***	0.02	(0.02, 0.03)	0.00	0.01	0.03	***
Processed	0.009	(0.008, 0.009)	0.003	0.006	0.009	***	0.44	(0.43, 0.46)	0.18	0.34	0.60	***
Ultra-processed (UPF)	0.026	(0.025, 0.026)	0.017	0.024	0.031	***	1.24	(1.22, 1.25)	0.90	1.19	1.51	***
**Drinks**												
Unprocessed or minimally processed (MPF)	0.038	(0.037, 0.040)	0.009	0.020	0.053		0.63	(0.62, 0.65)	0.28	0.53	0.85	
Processed	0.004	(0.004, 0.004)	0.000	0.000	0.004	***	0.23	(0.22, 0.25)	0.00	0.00	0.33	***
Ultra-processed (UPD)	0.006	(0.006, 0.007)	0.001	0.003	0.008	***	0.42	(0.41, 0.44)	0.07	0.27	0.60	***

^1^ Assesses the difference with the reference unprocessed or minimally processed food or drinks;

^*^ < 0.05, ** < 0.01 *** < 0.001

Compared with MPF, consumption of UPF contributes more to GHG emissions (36% vs 30%) (*p* < 0.001) but less to blue water use (19% vs 35%) (*p* < 0.001) per 2000 kcal. UPD determined approximately twice less GHG emissions (7% vs 12%) (*p* < 0.001) and seven times less blue water use (4% vs 27%) (*p* < 0.001) compared with MPD.

Dietary costs for UPF (€1.24/2000 kcal) and UPD (€0.42/2000 kcal) consumption were lower compared with costs of MPF (€1.32/2000 kcal) (*p* < 0.001) and MPD (€0.63/2000 kcal) (*p* < 0.001) consumption.

### Sensitivity analysis

In a sensitivity analysis, all bread was classified as processed instead of ultra-processed. The percentage UPF in ‘Grains and breads’ decreased from 76 to 35%. As a result, the average fibre content of UPF decreased with 0.2 g fibre per 100 g (2.1 g fibre per 100 g). Daily average UPF consumption decreased from 456 g per 2000 kcal to 336 g per 2000 kcal, resulting in an difference of 120 g (309 kcal). Obviously, UPF contributed less to daily intake of fibre (-6.3 g, -57%), protein (-12.6 g, -38%), sodium (-523 mg, -33%) and determined less GHG emissions (-0.14 kg CO_2_-eq, -8%), blue water use (-0.003 m^3^, -12%) and food costs (-€0.23, -19%).

## Discussion

This study investigated nutritional quality, environmental impact and costs of foods, drinks and daily diets according to the degree of processing across the Dutch population. Per 100 g, ultra-processed foods were on average energy-denser, less healthy, and associated with higher GHG emissions but lower blue water use and were cheaper than unprocessed or minimally processed foods. Per 100 g, ultra-processed drinks had on average a similar energy and sugar content, similar GHG emissions but lower blue water use and were more expensive compared with unprocessed or minimally processed drinks. In the current Dutch dietary pattern, UPFD consumption accounted for 29% of daily food consumption in weight per 2000 kcal and determined 61% of daily energy intake. Children consumed more UPFD per 2000 kcal, and especially UPD, compared with adults and older adults. The consumption of UPFD was found to be unhealthy given its significant contribution to the intake of nutrients such as sodium (72%), sugar (64%) and SFA (54%). The high UPFD consumption determined 45% of GHG emissions and 23% of blue water use. Furthermore, food costs related to UPFD consumption were lower since UPFD determined a smaller proportion of daily food costs compared with those unprocessed or minimally processed foods.

The UPFD consumption in the Netherlands is comparable to studies from the USA (58%) [23] and the UK (57%) [24] but higher compared with studies from Brazil [25], Chile [26] or Canada [27], ranging between 22 to 48% of daily energy intake. Differences might be explained by the NOVA classification of bread. Firstly, all bread in our study was classified as UPF since most bread is mass-produced nowadays and contains additives. Secondly, the Dutch consume large quantities of bread: on average 120 g or 309 kcal on a daily basis. Other studies sometimes categorized bread as unprocessed or minimally processed [28], processed [29] or ultra-processed [30]. The difficulty of classifying bread according to NOVA has been addressed previously as terminology such as artisanal bread, sliced or unsliced, mass-produced is used, but their exact interpretation is not self-evident [31, 32]. The classification of bread has direct implications for protein and fibre since we identified high – or similar compared to unprocessed or minimally processed foods – levels in UPF. In a sensitivity analysis we demonstrated that if 120 g or 309 kcal of UPF shifted to processed foods, consequently a lower contribution from UPF to daily protein and fibre intake was observed. This underlines a certain level of arbitrariness in food classification since results would be significantly different if bread was not classified as UPF. In accordance with previous studies, our overall results show that ultra-processed foods and drinks are unhealthy as they are on average more energy dense, contain high levels of SFA, sodium and sugar [33] and contribute significantly to daily energy, SFA, sodium and sugar intake [4].

We found that children and teenagers from 1 to 18 years consumed more UPFD than adults and older adults. This finding is in line with studies from Belgium [30], the USA [34], Canada [27] and Chile [26]. UPFD consumption appears to be inversely associated with age, as demonstrated in various studies [27, 29, 34, 35]. Our results indicated that the lower UPFD consumption with increasing age was mainly due to lower consumption of UPD. The older population consumed more unprocessed or minimally processed drinks such as coffee, tea and water, which raises the question of whether the observed UPD consumption in the younger age groups is a temporary effect or whether it is a birth-cohort effect that will remain when this groups reaches adult and older ages. Furthermore, although children do not consume 2000 kcal daily, the relative observed dietary share of UPFD for children and teenagers (1 to 18 years) in our study (75% of daily energy intake (not standardized)) was higher than reported values from UK (65%) [36] or Belgium (33%) [30]. The high consumption of UPFD among Dutch children is of concern, given its association with poor diet quality, weight gain, obesity and other adverse health outcomes [37]. Convenience, attractiveness and aggressive marketing campaigns targeting children can increase UPF consumption in children and is suggested as an important reason why energy intake from UPF is high in high-income countries [3].

The environmental impact per kg foods or diets according to the degree of processing (NOVA) has not been determined in detail in previous research. Fardet and Rock (2020) demonstrated based on GHG emissions of dietary patterns, that UPF-like discretionary foods do not necessarily produce the highest GHG emissions (per 100 g) [5]. In our study, UPF, per 100 g, were associated with on average a higher GHG emission, but lower blue water use compared with MPF. Per 100 g, the environmental impact for UPD was on average similar for GHG emissions but lower for blue water use compared with MPD such as water, coffee, tea and fruit- and vegetable juices. Those results are divergent and do not convincingly reflect a lower environmental impact for MPFD, compared with UPFD. Moreover, our study showed that UPF and UPD consumption contributed, respectively, 36% and 7% of GHG emissions and 19% and 4% of blue water use. An Australian study estimated the environmental impact of discretionary food consumption and reported at 33% and 35% for associated GHG emission, and water footprint, respectively [38]. The environmental impact associated with UPFD consumption is significant and should therefore not be neglected [39].

Food costs according to NOVA were not in line with outcomes for nutritional quality, as healthier foods (MPF) were more expensive than UPF, while UPD were twice as expensive as MPD. Previous studies did not assess food costs separately for drinks and foods, but did report that UPFD were less expensive than MPFD, for instance, in Belgium (€0.55/100 kcal for UPF and €1.29/100 kcal for unprocessed or minimally processed food) [40], although there are exceptions [41]. Moreover, food costs associated with MPFD consumption were more expensive than costs associated with consumption of UPFD. In Belgium, MPFD contributed most to daily dietary costs (30–42%) compared with UPFD (22–30%) [40]. Higher food costs for unprocessed, healthier foods and diets, might have implications for population health, especially among the lower educated individuals [8, 9].

The applicability of categorizing foods for health-related outcomes according to NOVA is frequently addressed. In addition, the concept of NOVA is more often used in food education. Given current diet-related NCDs and progressive climate change, but also growing gaps in health inequalities and economic status, integrated measures not focussing on one single problem, such as dietary health via NOVA, are preferred. Furthermore, a clear diet advice requires that outcomes for nutritional quality, environmental impact and diet cost are ideally in accordance with each other, for foods as well as for drinks, to facilitate a transition. The NOVA classification could potentially be used to distinguish nutritional quality, environmental impact and cost of diets. NOVA seems suited to identify unhealthy foods, but there are some exceptions: the UPF category in NOVA covers a broad range of unhealthy but also nutritious foods (e.g. wholegrain bread). Although, NOVA was developed to identify degree of food processing, the concept appears not to be of added value for classification for environmental impact and costs evaluations. Our results show no convincing and rather divergent results based on the NOVA concept in assessing—besides nutritional quality—the environmental impact and food costs for UPFD compared with MPFD. Therefore, we question whether food classification according to the degree of processing (NOVA) is needed and a suited methodology to implement for environmental impact as well as for diet cost evaluations or to use as a starting point for food policy.

However, action is needed since current food consumption patterns include many UPFD and a large number of foods that are unhealthy and not recommended for a healthy diet. Interventions focussing on the replacement of UPFD with MPFD will benefit human health but may not automatically lead to a lower environmental impact or reduced food costs. However, for example, replacing sugar-sweetened beverages with tap water is less expensive and benefits both human and planetary health [42]. For population groups with a general overconsumption, reducing UPF consumption without substituting recommended healthy foods remains an interesting lever for achieving a healthier and sustainable diet without adverse health effects [5]. It should be noted that it can be difficult to reduce UPFD consumption, as they are integrated into the diets of many consumers and should be part of an sustainable food system [5, 6]. Therefore, if we continue to consume UPF, it is worthwhile to explore the possibilities to reformulate UPF and UPD in such a way that at least their nutritional composition benefits human health by a lower SFA or sugar content while reducing environmental impact.

### Strengths and limitations

This is the first study to explore nutritional quality, environmental impacts and costs of foods and drinks and their consumption, according to the NOVA classification. A significant strength of this study is the quantification of the environmental impact in terms of GHG emissions and blue water use of UPFD and their consumption, using a comprehensive set of environmental indicators. The Dutch LCA Food database provides data on the most frequently consumed Dutch foods and covers 75% of the Dutch diet in weight. GHG emissions were used as a proxy for some other indicators available in the LCA Food database, however, we did not include other sustainability aspects such as animal welfare or pesticide use. The current study has several noteworthy limitations. Firstly, we used memory-based food consumption data, which is associated with misreporting, underreporting or overreporting of dietary intake [43]. Therefore, the reported food consumption by the degree of processing might over or underestimate the true levels. Secondly, UPFD were determined using the NOVA classification. NOVA is the most used system to classify foods by level of processing and is widely recognized as a tool for research into nutrition and public health. However, it should be noted that classification systems such as NOVA conceptually differ from processing level concepts in food science technology [44]. The use of NOVA enables comparison with other studies. Nevertheless, different definitions of UPFD and insufficient standardization make food classification with NOVA difficult [45] and can lead to confusion and subjective recoding of national food consumption databases [32]. Although we had to make assumptions as well, food consumption data in our study was collected with a great level of detail and systematically stored [16]. We were, therefore, able to systematically categorize foods according to NOVA with little inconsistencies or subjective classifications. Thirdly, when interpreting the results and comparing them with other studies, it is important to consider the expression of UPFD consumption. We expressed UPFD consumption as g per kcal to account for total energy intake. Moreover, as light beverages do not contain energy, actual UPD consumption is likely to be underestimated when exclusively using percentage of energy intake. Nonetheless, sensitivity analyses using the energy percentage of UPFD were carried out and did not alter our conclusions. Finally, estimated diet cost might be underestimated because only the lowest prices were included in the Dutch food price database. The food price data used was collected in the past (2017) to reflect prices from when dietary data was collected (2012–2016) and may differ from prices today due to inflation or VAT increasing from 6 to 9% for foods and beverages in the Netherlands. Although absolute food costs may differ, this method is suitable for the purpose of ranking foods based on total dietary cost.

## Conclusion

With this study we provide insight into the associated nutritional quality, environmental impact and costs of foods, drinks and daily diets according to the degree of processing across the Dutch population. Compared with unprocessed or minimally processed foods and drinks, UPF and UPD were found to be less healthy considering their high energy, SFA, sugar and sodium content. However, UPF were associated higher GHG emissions and with less blue water use and food costs. Therefore daily blue water use and food costs might increase if UPF are replaced by those unprocessed or minimally processed. As nutritional quality, environmental impacts and food costs relate differently to the NOVA classification, the classification is not directly applicable to identify win–win-wins of nutritional quality, environmental impacts and food costs. However, given the current high consumption of UPFD, especially in children, a lower consumption would reduce unhealthy intakes of energy, SFA, sugar and sodium, as well as avoid unnecessary GHG emissions.

## Supplementary Information

**Addtional file 1:Supplementary table 1. **Food group category rules for assessment of degree of processing.

## Data Availability

The datasets used during the current study available from the corresponding author on reasonable request.
