# Supplementary material for: Evaluation of foods, drinks and diets in the Netherlands according to the degree of processing for nutritional quality, environmental impact and food costs
Source: BMC Public Health. 2022 May 3;22:877. doi: 10.1186/s12889-022-13282-x (PMC9063197; doi:10.1186/s12889-022-13282-x)
Supplement: Supplementary file 1 — Addtional file 1:Supplementary table 1. Food group category rules for assessment of degree of processing. [file 12889_2022_13282_MOESM1_ESM.docx]

**SUPPLEMENTARY TABLES**

**Supplementary table 1.** Food group category rules for assessment of degree of processing.

**Supplemental table 1.** Food group category rules for assessment of degree of processing

| **Food group** | **Unprocessed or minimally processed foods and drinks** | **Processed culinary ingredients** | **Processed foods and drinks** | **Ultra-processed foods and drinks** |
| --- | --- | --- | --- | --- |
| **Potatoes and other tubers** | Conservation method is fresh, vacuum, frozen, dried, home-made, unknown |  | Potato product frozen or vacuum with added fat, salt or marinated | Manually, based on Dutch food names: Ready-to-eat potato products such as potato croquette, pommes duchesse |
| **Vegetables** | Conservation method is unknown, frozen, dried, fresh, vacuum, canned or jarred. |  | Conservation method is marinated, canned or jarred with added sugar, salt or fat, or unknown | Manually 'onions, deep fried, dried', ‘atjar tjampoer’ and frozen vegetables ready-to-eat and include industrial formulations (spinach a la crème etc) |
| **Legumes** | Conservation method is unknown, frozen, dried, fresh, vacuum, canned or jarred. |  | Conservation method is marinated, canned or jarred with added sugar, salt or fat, or unknown |  |
| **Fruits and olives, fruit compote** | Conservation method is not known, frozen, dried, fresh, heat treated, canned, jarred or medium is in water. |  | Conservation method is marinated, confit,  jarred or canned with added sugar, salt or fat, and medium is in syrup, juice or unknown | Industrially prepared |
| **Nuts, seeds and nut spread** | Unsalted or if salt content is not specified |  | Salted, sugared | If peanut spread and include industrial formulations |
| **Dairy** | Not sweetened, not specified if sweetened. |  | If added sugar, salt or fat. | If sweetened, additives, colours, emulsifiers added |
| **Cheeses** |  |  | If not spreadable | If spreadable |
| **Cream desserts, puddings (milk based) and Ice cream and substitutes, sorbet and water ice** |  |  |  | All foods are categorized as UPF |
| **Dairy and non-dairy creams, creamers** | If not sweetened |  | If sweetened for dairy creams and creamers | If sweetened for non-diary creams and creamers |
| **Flours, starches, flakes, semolina** | All un-or minimally processed |  |  |  |
| **Pasta, rice, other grain** | If conservation method is fresh, not known, dried, vacuum. If pasta is unfilled. |  |  | If conservation method is canned or precooked/frozen and include industrial formulations. If filled pasta.  Instant noodles are categorized manually. |
| **Bread, cripsbread, rusks, cough and pastry (plain puff, short-crust)** |  |  |  | All foods are categorized as UPF |
| **Breakfast cereals** | If sweetened is not specified |  |  | If sweetened or it was specified as unknown if sweetened (default is sweetened) or include industrial formulations. |
| **Meat, meat products and substitutes** | If conservation method is fresh, not specified, frozen or vacuum. |  | If conservation method is canned or jarred, dried, salted, smoked, marinated | Meat including industrial formulations |
| **Processed meat** |  |  | Manually: Processed foods may contain additives used to preserve their original properties or to resist microbial contamination, such as ham, bacon, pastrami and similar. | Manually: meats for which processes include hydrogenation, hydrolysis, extruding, moulding, reshaping, pre-processing by frying, baking; meat containing additives not used to preserve or to resist microbial contamination; pre-prepared meat and other reconstituted meat, such as nuggets en sticks, sausages, burgers, hot dogs, cordon blue. |
| **Meat substitutes** |  |  |  | All foods are categorized as UPF |
| **Fish, crustaceans, mollusc, amphibians and reptiles** | If conservation method is fresh or frozen or unspecified, vacuum, canned/jarred, in water (medium). |  | If conservation method is canned or jarred and medium is not known or in oil; Marinated, smoked/salted fish |  |
| **Fish in crumbs** |  |  |  | All foods are categorized as UPF |
| **Eggs and egg products** | All foods are categorized as un- or minimally processed |  |  |  |
| **Fats and oils** |  | Vegetable oils, butter, animal fats |  | Margarines and cooking fats (mixed) |
| **Sugar, honey, jam, syrup, sweet sauce** |  | Sugar, honey |  | All foods are categorized as UPF, except for the processed culinary ingredients. |
| **Cakes and sweet biscuits** |  |  |  | All foods are categorized as UPF |
| **Fruit and vegetable juices** | If unsweetened |  | If sweetened |  |
| **Carbonated/soft/isotonic drinks, diluted** |  |  |  | All foods are categorized as UPF |
| **Coffee, tea and herbal teas** | If tea unsweetened, coffee unsweetened, with milk |  |  | If iced coffee sweetened, powdered or instant coffee  tea, powdered(instant) or iced coffee ready to eat or and include industrial formulations. |
| **Waters** | Plain waters |  |  | If additives added /sugared or and include industrial formulations. |
| **Alcoholic beverages** |  |  | Wine, ciders, fruit wines, sherry, porto, vermouth, beer | Spirits, brandy, Aniseed drinks (pastis) , Liqueurs, mixed punches, cocktails |
| **Condiments, spices, sauces and yeast** | Fresh | Vinegars | If ‘home-made’ sauces without ingredients being disaggregated | If consistence is powder or concentrate. Industrial prepared sauces or sauces with unknown preparation method. |
| **Soups and stocks** |  |  |  | All foods are categorized as UPF |
| **Vegetarian products** |  |  |  | If industrial prepared |
| **Dietetic products** |  |  |  | Artificial sweeteners, meal replacers (shakes, bars, powders): all categorized as UPF |
| **Insects** | All foods are categorized as un- or minimally processed |  |  |  |
| **Savory snacks** |  |  |  | All foods are categorized as UPF |
